# Definitions of parasitism, considering its potentially opposing effects at different levels of hierarchical organization

**DOI:** 10.1017/S0031182023000598

**Published:** 2023-08

**Authors:** Lajos Rózsa, József Garay

**Affiliations:** 1 Institute of Evolution, Centre for Ecological Research, Budapest H-1121, Hungary; 2 Centre for Eco-Epidemiology, National Laboratory for Health Security, Budapest, Hungary

**Keywords:** costs of parasitism, definition of parasitism, host population growth, levels of biological organization, multilevel selection

## Abstract

An annotated synthesis of textbook definitions of parasitism is presented. Most definitions declare parasitism is a long-lasting relationship between individuals of different species harming the hosts. The infection-induced costs are interpreted as diseases in the medical-veterinary literature. Alternatively, evolutionary ecologists interpret it as a reduction of host's fitness (longevity, fertility or both). Authors often assume that such effects decrease host population growth and select for antiparasitic defences, which is not necessarily true because infections may simultaneously express opposite effects at different levels of biological organization. (i) At the cellular level, infection-induced cell growth, longevity and multiplication may yield tumours maladaptive at higher levels. (ii) At the individual level, reduced host longevity, fertility or both are interpreted as disease symptoms or reduced fitness. (iii) Contrary to common sense, the growth rate of infected host lineages may increase in parallel with the individuals' reduced survival and fertility. This is because selection favours not only the production of more offspring but also their faster production. (iv) Finally, infections that reduce host individuals' or lineages' fitness may still increase infected host populations' growth rate in the context of ecological competition. Therefore, differences between parasitism and mutualism may depend on which level of organization one focuses on.

## Graphical abstract

**Figure:**
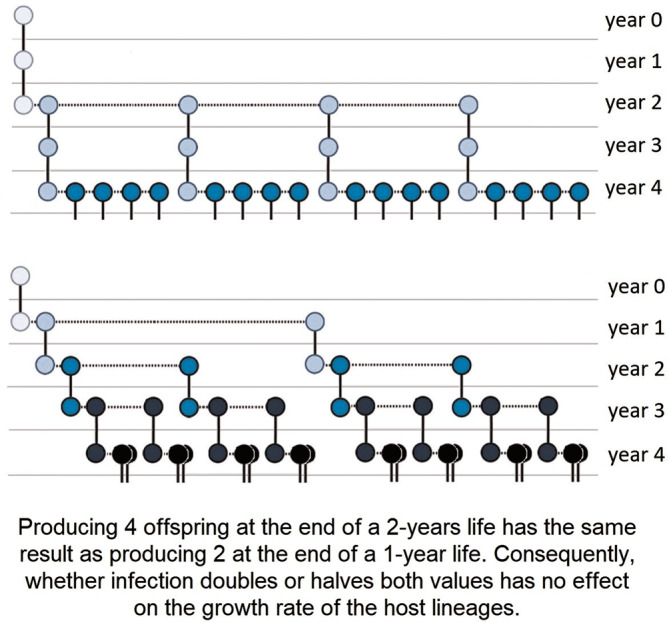

## Definitions of parasitism and parasites

Definitions are statements on the meaning of concepts and terms; they are arbitrary tools created to allow for scientific understanding. Definitions need to be operational (applicable), although different purposes often require slightly different definitions of the same concept, and therefore absolute unanimity is not necessarily sought. Nevertheless, certain constancy is still needed to maintain meaningful communication. Definitions that literally mean something other than their intended meaning, or those that are ambiguous in meaning, can be considered erroneous.

A definition of parasitism is essential in teaching parasitology and in textbooks to summarize the general features – so-called ‘laws’ (Poulin, 2007*a*) – of parasitism, particularly to specify the range of validity of the statements, hypotheses and predictions. Unfortunately, standard textbook definitions of parasitism are highly inconsistent or contradictory (Zelmer, 1998). Almost all authors agree that parasites harm their hosts by utilizing them as nutrient resources; however, further details can be desperately confusing.

First, a compilation of the main points of the evolutionary-ecological definitions of parasitism is provided below. This is based on the fragmentary definitions in the available literature (Rothschild and Clay, 1952; Anderson and May, 1978; Price, 1980; Olsen, 1986; Barnard, 1990; Rohde, 1993; Clayton and Moore, 1997; Poulin and Morand, 2000; Combes, 2001; Ebert, 2005; Nunn and Altizer, 2006; Poulin, 2007*b*; Leung and Poulin, 2008; Martin and Schwab, 2013; Goater *et al*., 2014; Locker and Hofkin, 2015; Clayton *et al*., 2016; Lucius and Poulin, 2017; Schmid-Hempel, 2021; Kaishian *et al*., 2022). Next, the most crucial criterion is scrutinized; that parasites harm their hosts. Obviously, the usual interpretation of the meaning of ‘damage’ (or ‘cost’) is often contradictory. Finally, no single ‘superior’ definition is proposed to resolve the controversy; only ideas for further consideration and discussion are provided.

## Parasitism as a form of symbiosis: a compilation

### Symbiosis

Symbiosis is an ecological relationship realized through a direct body-to-body interaction between individuals of separate species, including mutualism and parasitism. Accordingly, intraspecies exploitative interactions do not qualify as parasitism. The dwarf males of the deep-sea anglerfish (Ceratioidei) obtain nutrients from the much larger females, to which they are permanently attached. These males are not parasites, even if they are often called ‘parasitic males’ (Pietsch, 2005). Likewise, the exploitation of some people by others does not constitute parasitism, although colloquialism sometimes uses the term in this context (see e.g. Lunsing, 2003), which may seem offensive and unethical to many (Zimmer, 2000). Further, this criterion excludes associations where the target of exploitation is not a host individual but a group of hosts (like family, colony, etc.). Thus, brood-parasitic birds which exploit host families (Soler, 2018), and social parasites which exploit colonies of eusocial insects (Hölldobler and Kwapich, 2022) are all excluded, limiting the concept to individual-level exploitative interactions between species (but see also Rothschild and Clay, 1952; Barnard, 1990; Payne, 1997).

### Intimate relationship

Parasite individuals live in long-standing body-to-body contact with host individuals; inhabiting a single host individual at least for a whole phase of the parasite developmental cycle but often through several life cycles (Price, 1980; Nunn and Altizer, 2006; Poulin, 2007*a*, 2007*b*; Locker and Hofkin, 2015; Clayton *et al*., 2016; Lucius and Poulin, 2017). This long-lasting nature of the bodily contact – colloquially called an ‘intimate relationship’ (Combes, 2001) – excludes ‘kleptoparasites’ from animal parasitism and grazers (locusts, ruminants, etc.) from the concept of phytoparasitism. Similarly, it excludes many blood-sucking insects (tabanids, mosquitoes, etc.), ‘micropredators’ in the sense of Poulin (2011), which have only fleeting contact with their hosts (Lehane, 2005).

### Hosts as habitat patches

From the above, it is obvious that the host body acts as a well-defined ‘habitat patch’ for the parasites (and symbionts) (Rothschild and Clay, 1952; Anderson and May, 1978; Price, 1980; Rohde, 1993; Clayton and Moore, 1997; Poulin, 2007*b*; Locker and Hofkin, 2015). This implies that they are relatively small, more numerous, have shorter life cycle and higher reproductive potential, while hosts are large, less numerous, have longer life cycle and lower reproductive potential.

### Nutritive relationship

Parasites, just like mutualists, obtain nutrients from the host body and excrete their metabolic by-products (potentially toxic or carcinogenic wastes) there. A similar but non-nutritive interaction is phoresy, which is an exploitation of host mobility and, consequently, host energy resources (Bartlow and Agosta, 2021).

### Antagonistic relationship

Parasitism is an antagonistic relationship that damages the host body structure, metabolism or both while benefiting the parasites. In addition, infections often cause further disadvantages, such as exclusion from social (Kurzban and Leary, 2001) and sexual relationships (Hamilton and Zuk, 1982), increased chance of predation (Hudson *et al*., 1992) and additional co-infections (Sepkowitz, 2002). Contrarily, some parasites may also induce certain beneficial side-effects, e.g. through reduced predation pressure (Hasik *et al*., 2023), through negative interactions with other, more virulent infections (Jenner, 1798; Locker and Hofkin, 2015), but their net effect on the host is negative by definition. This implies that parasites are dependent on their hosts, while hosts are non-dependent on parasites (Crofton, 1971*a*; Rohde, 1993). Consequently, parasitism is obligatory for the parasites, but occurrent to the hosts (Ebert, 2005).

### Host adaptations to avoid parasite transmission, survival and reproduction

Infections can drastically affect host populations (Hasik and Siepielski, 2022), even though a publication bias may cause mild and symptomless infections to appear much rarer than in reality. The infections' net effects on the hosts can even be so subtle that it is impossible to directly measure, prove or document them. However, these effects may still exert selective pressure on the host populations to evolve adaptive responses to avoid parasite transmission. Due to these host adaptations to avoid infections, transmission from one host to another is a hazardous episode of the parasite life cycle, most often leading to high parasite mortality (Dobson *et al*., 1992; Combes, 2001; Leung and Poulin, 2008; Locker and Hofkin, 2015).

Similarly, host populations frequently also evolve adaptive responses to defend against established infections, to hinder parasite survival, growth and multiplication, providing an indirect verification of the antagonistic nature of the relationship (Poulin, 2007*b*; Leung and Poulin, 2008). Despite their counter-adaptations, parasites' vulnerability to host defences typically constitutes a significant cause of their mortality (other than the losses during transmission, see above). Naturally, host nutrient and energy resources allocated to avoidance and defence constitute further parasite-induced metabolic losses.

Since phytophagous arthropods' most effective natural enemies are often species other than the host plants, like parasitoids and predators (Cornell and Hawkins, 1995; Hawkins *et al*., 1997), this argument does not necessarily apply to them. Therefore, it is advisable to exclude them (most of all species on Earth) from the concept of parasitism (but see also Price, 1980; Clayton *et al*., 2016).

Note that host adaptations to avoid or to fight an infection indicate that this symbiont has been parasitic in the past, but not necessarily in the present.

### Parasites' distribution across host individuals

Since parasite avoidance and antiparasitic defence are variable traits across hosts, the distribution of parasite individuals across host individuals is aggregated (overdispersed) (Crofton, 1971*b*; Anderson and May, 1978; Clayton and Moore, 1997). This means that most parasite individuals inhabit a few heavily infected hosts (those with poor avoidance and defence capabilities), while most of the host individuals (those with average or better capabilities) harbour few if any parasites. Contrarily, mutualists often express other types of frequency distributions across host individuals, such as even and polymodal distributions (see e.g. Britayev *et al*., 2007; Elliott *et al*., 2009).

### The parasitism–mutualism continuum

Contrary to parasites, symbionts which exert positive net effects to the hosts are called mutualists. According to the net costs or benefits caused to the hosts, there is a continuum from parasitism through neutral commensalism to mutualism. Host–symbiont species pairs may shift quite flexibly along this continuum, the nature of their relationship may vary through space and time (Bronstein, 1994; Leung and Poulin, 2008; Rózsa and Apari, 2012; Lucius and Poulin, 2017).

### Parasites do not usually kill their hosts

Parasites do not usually kill their hosts and therefore, they are not adapted to transmit from dead hosts to new ones. This differentiates them from parasitoids, which kill their host as a prerequisite for successful development and transmission to further host individuals (Rothschild and Clay, 1952; Anderson and May, 1978; Poulin and Morand, 2000; Ebert, 2005; Poulin, 2007*a*, 2007*b*; Goater *et al*., 2014; Locker and Hofkin, 2015; Lucius and Poulin, 2017). According to the different degrees of lethality caused by infections, there is a continuum from non-lethal (more or less mild) parasitism to lethal parasitism (properly called parasitoidism). Host–symbiont species pairs may shift quite flexibly along this continuum, the nature of their relationship may vary through space and time. Taking trophically transmitted parasites as an example, they often manipulate intermediate host behaviour to increase predation by the definitive host. This means that they kill their hosts indirectly through a predator, the frequency and effectiveness of which can vary between species.

### Taxonomic scope of parasitism

Finally, several authors specify a taxonomic scope of parasitism for historical and practical reasons (Poulin, 2007*a*, 2007*b*; Locker and Hofkin, 2015). Some authors delimit the concept to include only animals and ‘animal-like’ (eukaryotic and heterotrophic) protists that parasitize animals (e.g. Goater *et al*., 2014). Others include all types of microbes, plants and fungi, both as hosts and as parasites. Finally, some authors include even suborganismal entities – like viruses and macromolecules, such as nucleic acid or protein strands (RNA, DNA, prions) – into the concept of parasitism, setting the maximum of a taxonomical domain (Combes, 2001; Lucius and Poulin, 2017).

## Infection-induced harms across 4 levels of hierarchical organization

Whatever definition is preferred, the notion that parasites exert a net harmful effect on their hosts is a foremost characteristic of host–parasite relationships. From ancient times, this feature contributed much to the negative emotions about parasites (Zimmer, 2000; Kaishian *et al*., 2022). Contrarily, mutualists are defined by the net beneficial effect they exert on infected hosts.

It is traditional in the human and veterinary medical literature to treat infection-induced harms as a synonymy of parasites' pathogenicity, i.e. their capacity to produce disease, thus increasing the host population's morbidity and mortality rates (Vihinen, 2017). However, at the dawn of the science of parasite ecology and evolution, it became evident that practically all large-bodied organisms are infected by some (and often by several) species of micro- and macroparasites (i.e. species that are usually thought of as parasites) most often without ever developing symptoms of any disease. Thus, the notions of morbidity and disease had to be replaced by an evolutionary-ecological concept of the harms caused by parasites. For this reason, Anderson (1978) and Anderson and May (1978) opened a new avenue of definitions by proposing that a parasite causes an ‘inducement of host mortalities and/or reduction in host reproductive potential’ and, thus, exerts a ‘detrimental effect on the intrinsic growth rate of its host population’. The authors defined both birth and death rates as events/host/unit of time. Note that individual and population-level harms of infection are mentioned as if they were equal.

However, this idea ignores the nested hierarchical nature of the biological organization, which is a fundamental feature of life (Szathmáry and Maynard Smith, 1995). Therefore, the crucial question is whether or not infection-induced effects at different levels of the biological hierarchy are necessarily consistent with each other.

### Level 1: host cells

A multicellular organism's survival and reproductive success depend on its ability to regulate its cells' growth, survival and reproduction. Cellular infections can modify all these 3 components. First, programmed cell growth can be obstructed by various protist and fungal infections, most notably by Microsporidia, occurring predominantly in fish hosts. Infected cells grow into vast masses of parasites packed within a single host cell called xenoma (Lom and Dyková, 2005). Second, programmed cell death (apoptosis) and controlled cell division are also essential. Whenever cell division runs amok in an unregulated manner, a malignant tumour develops, threatening the very existence of the multicellular individuals. Taking the population of the USA as an example, approximately 3–4% of all cancer cases are attributable to the most common carcinogenic infections like *Helicobacter pylori*, hepatitis B virus, hepatitis C virus, human herpes virus type 8, human immunodeficiency virus or human papillomavirus (Islami *et al*., 2018). Evidently, infection-induced increase in cellular growth, survival and reproduction reduces the survival and reproduction of the multicellular host organism.

### Level 2: host individuals

For obvious reasons, medical and veterinary scientists focus their interest on diseases. Symptoms define diseases, and – understandably – infected hosts' reduced lifespan and fertility are among the most obvious symptoms. Contrarily, health is characterized by a long and fertile life. Therefore, infections that increase host longevity, fertility or both are classified as mutualists. Similarly, in the evolutionary-ecological literature, a parasite-induced reduction of host lifetime reproductive success (LRS) is also interpreted as indisputable evidence for parasitism by thousands of research articles (see e.g. Herre, 1993; Lipsitsch *et al*., 1996; van Dijk and De Baets, 2021).

### Level 3: host lineages

Lineages are comprised of host individuals linked by genetic inheritance along the parent–offspring relationships. Lineages are usually not regarded as a distinct level of biological organization; however, ever since Darwin (1859) they represent units of selection (see e.g. Garay *et al*., 2016; Nunney, 2019, Krishnan *et al*., 2023). In modern days, the concept of multilevel selection posits that selection occurs in parallel at various levels in the hierarchy of biological organization (Wade *et al*., 2010). Following these views, lineages are treated here as one of the parallel levels of selection.

For sake of simplicity, we consider only parthenogenetic (asexual) host lineages. At first glance, this appears to be nonsense because a symbiont which transmits exclusively along mother-to-daughter routes in a parthenogenetic host population necessarily tends to a mutualist way of life. Otherwise, if it reduces host fitness, then it will be lost from the host population (Fine, 1975; Frank, 1996). In this case, however, the growth rates of infected and non-infected host lineages must be compared. For this purpose, it is not necessary to presume that parasites or mutualists are transmitted along these lineages; instead, the genetic predispositions of hosts (such as genes regulating the immune system) are transmitted along the parent–offspring relationships. Thus, whatever transmission route (horizontal or vertical) is used by parasites, it is the host's infection state (infected or uninfected) that is inherited along the lineages.

By definition, parasites are expected to reduce the growth rate of infected lineages compared to uninfected ones, while mutualists are expected to exert the opposite effect. Readers may intuitively assume that infections which decrease the host individuals' survival and reproduction necessarily decrease the fitness of infected host lineages too – which is not necessarily true. Fitness is a term with several subtly different meanings (Orr, 2009). Generally speaking, it is the expected contribution of alleles, genotypes or organisms to future generations relative to the other alternative alleles, genotypes or organisms in the population (Stearns, 1992; Pásztor *et al*., 2016). Let us consider a hypothetical example. Part of the population harbours an infection that halves both longevity and fertility of infected hosts. Say, uninfected hosts complete their life cycle in 2 years and produce 4 offspring at the end of their life, while infected hosts live only for 1 year and produce only 2 offspring at the end of their life. Thus, the annual growth of the uninfected host lineage is 1, 1, 4, 4, 16 through 5 years, while that of the infected hosts is 1, 2, 4, 8, 16 through the same period. In this imaginary case, infection halves both the longevity and LRS of host individuals, while it does not change the fitness (growth rate) of infected lineages. Logically, this symbiont should be classified as a neutral commensal. Under the same simplistic circumstances, a symbiont that doubles the lifespan and fertility of infected hosts would also not qualify as a mutualist.

**Figure 1.**
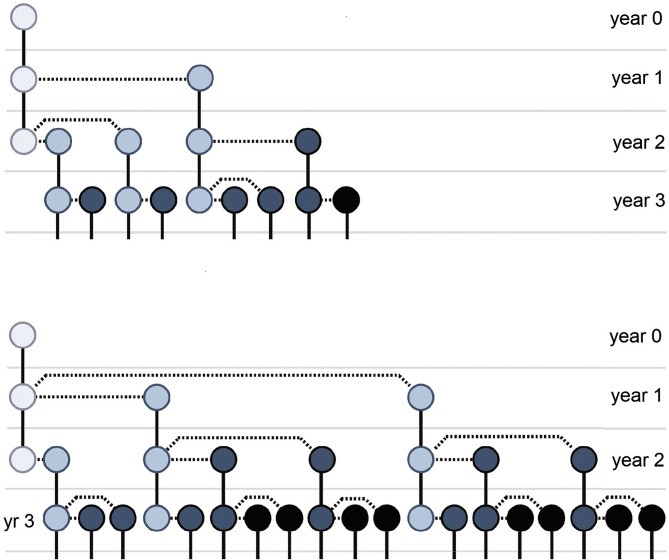
An infection that does not change host lifespan, lifetime reproductive success (LRS) or mean annual fecundity may still increase or decrease the growth rate of host lineages. Representation of 2 hypothetical parthenogenetic host lineages, where individuals' longevity is 3 years, there is no reproduction in the 1st year, and a total of 3 offspring are produced during the 2nd and 3rd years. Above, individuals breed less intensively in the 2nd year and more intensively in the 3rd year, which is reversed below. Shifting the reproduction peak to an earlier period in the life cycle increases growth rate, even though population dynamic indices (longevity, LRS, annual mortality and fecundity rates) are equal. Solid lines represent the survival of individuals to the following year, and dotted lines represent reproduction. Darker circles represent later generations than lighter ones (see also Box 4).

Real-life host populations, however, most often have overlapping generations; thus, the combined effects of changes in longevity and fecundity are more challenging to assess. Nevertheless, it is easy to construct hypothetical models in which reducing host lifespan, reproduction or both comes together with increasing host lineage growth. Examples are provided for infections that (i) reduce host longevity, do not affect LRS, but increase host lineage growth (Box 1), (ii) do not affect mortality, reduce LRS, but increase host lineage growth (Box 2), (iii) reduce both host lifespan and LRS, but increase host lineage growth (Box 3). In the last hypothetical example, not only host lifespan and LRS, but also the mean annual fecundity is unchanged, while the host lineage growth increases (Box 4). Note that all these counter-intuitive effects are based on the fact that selection favours not only the greater fecundity but also, the faster speed of producing offspring (Brommer *et al*., 2002), as visualized in Fig. 1.

Box 1. Increasing host mortality may increase host lineage growth

In population ecology, Leslie matrices are discrete-time, age-structured demographic models of populations with overlapping generations. They describe the multiplication of female individuals only. Let us consider a simple life history model where:

- the lifespan of the uninfected hosts is 3 years, and the 1st year juveniles do not multiply,
- the survival rate in the *t*-th year age is denoted by *ω*_*t*_,
- the average fecundity in the *t*-th year is denoted by *α*_*t*_,

thus the lifetime reproductive success (LRS) is *ω*_1_*α*_2_ + *ω*_1_*ω*_2_*α*_3_ (a host can reach the 2nd year with probability *ω*_1_ when it multiplies by *α*_2_, and the 3rd year with probability *ω*_1_*ω*_2_when it multiplies *α*_3_).

Using this notation, the Leslie matrix reads as
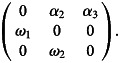

In this model, there are 3 ways how a symbiont might affect the infected host population:

- decrease or increase the life span,
- decrease or increase at least one survival rate,
- decrease or increase at least one fecundity rate.

The long-term growth rate (*R*_0_) of host lineages can be determined as the asymptotic growth rate (the dominant, positive eigenvalue of the Leslie matrix) corresponding to the stable age distribution (Caswell, 2001).

Using these notations, consider an infection that increases host mortality, does not affect LRS, but increases host lineage growth. Let us compare the Leslie matrices of infected *vs* uninfected host lineages of a hypothetical population. We assume that not the parasites themselves, but the hosts' state of being infected *vs* uninfected is transmitted through generations.

Uninfected hosts, LRS: 1 × 1 + 1 × 1 × 3 = 4
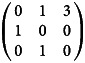
Infected hosts, LRS: 1 × 4 = 4
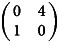

The long-term growth rate of the uninfected lineage is *R*_0_ ≈ 1.67, and that of the infected lineage is *R*_0_ = 2.

Box 2. Reducing host LRS may increase host lineage growth

Using the same notations and assumptions as above, consider the following example:

Uninfected host, LRS: 1 × 1 + 1 × 1 × 5 = 6
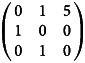
Infected host, LRS: 1 × 3 + 1 × 1 × 2 = 5
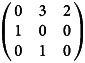

The long-term growth rate of the uninfected hosts is *R*_0_ ≈ 1.90, and that of the infected hosts is *R*_0_ = 2.00.

Box 3. Reducing host lifespan and LRS may increase host lineage growth

Using the same notations and assumptions as above:

Uninfected host, LRS: 1 × 1 + 1 × 0.55 × 2 = 2.1
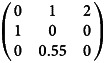
Infected host, LRS: LRS = 1 × 2 = 2
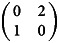

The long-term growth rate of the uninfected lineage is *R*_0_ ≈ 1.35, and that of the infected lineage is *R*_0_ ≈ 1.41.

Box 4. Host lifespan, LRS and mean annual fecundity are unchanged, but host lineage growth increases

Using the same notations and assumptions as above:

Uninfected host, LRS: 1 × 1 + 1 × 1 × 2 = 3
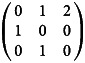
Infected host, LRS: 1 × 2 + 1 × 1 × 1 = 3
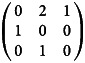

The long-term growth rate of the uninfected hosts is *R*_0_ ≈ 1.52, and that of the infected hosts is *R*_0_ ≈ 1.62 (see also Fig. 1).

To the best of our knowledge, no one has raised these points before, so previous studies have never interpreted the differences between parasite-infected and non-infected host lineages along these lines. However, the contradictory results of some studies seem to suggest that similar phenomena may occasionally occur. For example, fungal endophytes increase fecundity in the grass *Agrostis hyemalis* at the expense – or benefit? – of reducing its longevity (Yule *et al*., 2013). *Toxoplasma gondii*, a widespread infection in rodents and humans, increase predation pressure (Berdoy *et al*., 1995) and traffic accidents (Gohardehi *et al*., 2018) of infected hosts while, on the other hand, makes them sexually more attractive potentially increasing their reproductive success (Dass *et al*., 2011; Borráz-León *et al*., 2022). We cannot quantify the combined fitness effects of reduced lifespan and (presumably) increased reproductive success of infected hosts; however, host adaptations to combat *Toxoplasma* infections indicate that it is unfavourable at the lineage level.

### Level 4: host populations

We usually expect parasite infections to decrease and mutualist infections to increase the host population growth rate (Anderson and May, 1978). In real life, however, host populations are embedded in ecological communities formed by a network of several interspecific relationships. Relationships other than the host–parasite relationship in focus may influence the actual parasite-induced harm or benefit to the host population. Thus, certain symbionts that decrease host fitness at the former levels may still increase host population growth rate.

Looking at a multispecies community, successfully invading populations may benefit from hosting pathogens and transmitting them to endemic competitor populations (McNeill, 1976). ‘McNeill's Rule’, i.e. a pathogen-mediated negative interaction between host species (or populations) is also called ‘parasite-mediated competition’ or ‘apparent competition’ or ‘novel weapon hypothesis’ in the parasitological literature (Hudson and Greenman, 1998; Ricklefs, 2010; Vilcinskas, 2015). For instance, in human history, the spillover of smallpox and measles from the colonizing European populations decreased the competitive abilities of native populations (McNeill, 1976; Diamond, 1998). Similarly, apparent competition mediated *via* shared parasites contributes to the invasion success of introduced exotic gamebirds (Tompkins *et al*., 2002*a*), crayfish (Small and Pagenkopp, 2011) and squirrels (Tompkins *et al*., 2002*b*). This phenomenon is caused by the differential virulence (low in the coevolved invasive species and high in the naive indigenous hosts) that yields a parasite-mediated competitive advantage of the invasive population. Thus, an infection-induced reduction of individual fitness within the invasive host population comes together with the increased competitive success of this population.

Examples of the opposite scenario may also occur, i.e. symbionts that are mutualistic at the individual or lineage level but harmful at the population level. We suspect that *Wolbachia* infections of some insect populations may exemplify this case. Some strains of *Wolbachia* induce parthenogenesis in the infected insects, often making whole populations or species parthenogenetic (Knight, 2001). Switching to parthenogenesis likely increases individual and lineage multiplication rates since all the metabolic and genetic costs of sex are spared. However, parthenogenetic reproduction is a self-destructive strategy; asexual populations and species are more likely to go extinct in the long run (Moreira *et al*., 2021).

## Discussion

In this review, we first put the pieces of the textbooks' fragmentary descriptions together to compile an annotated definition of parasitism and parasites. Points 1–4 of this definition outline symbiosis (including both mutualism and parasitism) as a long-lasting, nutritive, body-to-body relationship between 2 individuals belonging to separate species. The 5th point posits that parasitism – unlike mutualism – is harmful for the host and beneficial for the parasite individual. The next 2 points are evolutionary-ecological consequences of this asymmetry postulating that hosts are adapted to avoid infections and to fight established ones, which results in an aggregated distribution of parasites. Hence, the antagonistic nature of this relationship is crucial in defining parasitism and parasites.

However, whether a relationship is mutually beneficial (mutualism) or harmful to one of the partners (parasitism) depends on which level of biological organization we focus on. Therefore, we overviewed the infection-induced harms (also known as costs, expenses, losses or damages) to the hosts at different levels. We have shown that negative effects expressed at a given hierarchy level can accompany positive effects at another hierarchy level. We do not claim that infections' opposing effects at different levels of organization would be a common phenomenon, neither that it would often cause problems in defining parasitism and parasites. We simply say that these phenomena are worth thinking about.

The fact that certain cellular infections can increase host cell growth, survival and reproduction, and thus give rise to malignant tumours fatal to host individuals is commonplace medical knowledge. In this case, an increase in the fitness of the infected cell line causes a decrease in the fitness of the host individual. This apparent contradiction does not cause any confusion in the definition of parasitism because the decrease in fitness is traditionally interpreted for individuals.

At the same time, the parasitological literature does not recognize the fact that a reduction in the lifespan of an individual does not necessarily mean a cost (harm) to the infected host lineages. This is because medical and veterinary scientists necessarily apply a ‘health-or-disease’ perspective, and living a longer life seems healthier. Contrarily, however, assuming LRS is equal, faster life cycle completion is adaptive compared to a slower and longer life. Thus, comparing the adaptive success of host lineages, higher mortality can outcompete lower mortality.

Similarly, higher LRS seems healthier than lower. However, assuming longevity is equal, we conclude that the pure number and quality of descendants are not the only factors influencing adaptive success. Selection also favours the speed of reproduction, thus an infection that makes hosts breeding in an earlier phase of the life cycle may be advantageous at the level of host lineages. For this reason, lineages with higher LRS can be outcompeted by lineages with higher long-term growth rate (*R*_0_) (Garay *et al*., 2016).

Although we do not deny the possibility of multilevel selection, we believe that the vast majority of antiparasitic adaptations (point 6 of the definition above) arise due to selection occurring at the lineage level. Admittedly, this is an ‘orthodox Darwinian’ viewpoint. The most obvious difference between the medical-veterinary (focusing on individual fitness) and the orthodox Darwinian (focusing on lineage fitness) perspectives lies in evaluating the significance of changes in host lifespan. From the former perspective, the reduced lifespan of the host (indicated by increased mortality) is the cost of infection; from the latter perspective (all else being equal), the reduced lifespan represents a faster life cycle, i.e. an advantage.

We have primarily used hypothetical examples to illustrate the complex way in which longevity, the number of descendants and the timing of their production determine the adaptive success or failure of lineages, with a few real-life examples also mentioned.

Finally, the fact that invasive host populations may benefit from carrying pathogens (even if they are harmful at the individual and lineage levels) in the context of ecological competition has been known for ages. Ironically, this ecological hypothesis was first introduced by a historian (McNeill, 1976) who recognized the importance of pathogen spillover from European invaders to indigenous people during the colonization of the Third World. Unfortunately, parasite ecologists still have trouble distinguishing between McNeill's rule (which postulates that the presence of certain infections is beneficial to invasive populations) and the Enemy Release Hypothesis (which postulates the opposite; that invasive populations benefit from the absence of certain infections). Rózsa *et al*. (2015) argued that these 2 hypotheses are not mutually exclusive but rather they tend to co-occur with 2 different types of invasions (invasions by small and isolated populations established by a few pioneers *vs* mass invasions). Nevertheless, ecologists still tend to treat McNeill's rule as a peculiarity, an exceptional situation when parasites confer benefits to the host populations. Probably due to the dominance of the traditional individual-focused viewpoint, community ecologists have not yet asked why to categorize these symbionts as parasites.

## Conclusion

Overall, we have shown that definitions of parasites and parasitism rely (among others) on the antagonistic nature of this relationship. However, the harms and benefits caused by parasites may be opposing across 4 different levels of organizational hierarchy making the differentiation between parasitism and mutualism vaguer than formerly thought. We believe that medical and veterinary parasitologists continue to focus on host individuals. Evolutionary biologists go on focusing on host lineages, because this is the hierarchical level where selection most effectively yields in adaptations. Finally, community ecologists are interested in the dynamics of host populations. Clarifying the differences between these viewpoints may hopefully help to increase understanding in communication across these different fields of science.

## Data Availability

No additional data are associated with this study.
